# Inversion of Left Ventricular Axial Shortening: In Silico Proof of Concept for Treatment of HFpEF

**DOI:** 10.3390/bioengineering11070676

**Published:** 2024-07-02

**Authors:** Wolfgang A. Goetz, Jiang Yao, Michael Brener, Rishi Puri, Martin Swaans, Simon Schopka, Sigrid Wiesner, Marcus Creutzenberg, Horst Sievert, Ghassan S. Kassab

**Affiliations:** 1 Cardiothoracic Surgery, University Hospital Regensburg, 93053 Regensburg, Germany; wolfgang1.goetz@klinik.uni-regensburg.de (W.A.G.); simon.schopka@klinik.uni-regensburg.de (S.S.);; 2 Dassault Systémes, Johnston, RI 02919, USA; jiang.yao@3ds.com; 3 Division of Cardiology, Columbia University Irving Medical Center, New York, NY 10027, USA; 4 Cleveland Clinic, Cleveland, OH 44195, USA; 5 St. Antonius Ziekenhuis, 3435 Nieuwegein, The Netherlands; 6 CardioVascular Center, 60389 Frankfurt, Germany; 7 California Medical Innovations Institute, San Diego, CA 92121, USA

**Keywords:** finite element method, computational simulation, aortic stiffness, atrio-ventricular plane displacement, ventricular strain, ventricular function, left ventricular apex, inverse left ventricular shortening, HFpEF

## Abstract

Left ventricular (LV) longitudinal function is mechanically coupled to the elasticity of the ascending aorta (AA). The pathophysiologic link between a stiff AA and reduced longitudinal strain and the subsequent deterioration in longitudinal LV systolic function is likely relevant in heart failure with preserved ejection fraction (HFpEF). The proposed therapeutic effect of freeing the LV apex and allowing for LV inverse longitudinal shortening was studied in silico utilizing the Living Left Heart Human Model (Dassault Systémes Simulia Corporation). LV function was evaluated in a model with (A) an elastic AA, (B) a stiff AA, and (C) a stiff AA with a free LV apex. The cardiac model simulation demonstrated that freeing the apex caused inverse LV longitudinal shortening that could abolish the deleterious mechanical effect of a stiff AA on LV function. A stiff AA and impairment of the LV longitudinal strain are common in patients with HFpEF. The hypothesis-generating model strongly suggests that freeing the apex and inverse longitudinal shortening may improve LV function in HFpEF patients with a stiff AA.

## 1. Introduction

Research indicates that atrioventricular plane displacement and LV longitudinal shortening are the primary contributors to heart pumping, accounting for 60% of the LV stroke volume and 80% of the right ventricular (RV) stroke volume [1]. Since LV longitudinal shortening is the major contributor to the heart’s stroke volume [2], any alterations in AA elasticity and the subsequent increase in mechanical load on the LV may play an relevant role in heart failure, particularly for heart failure with preserved ejection fraction (HFpEF). During the systolic longitudinal shortening of the heart, the atrio-ventricular plane, which includes the aortic annulus, is displaced towards the apex of the heart by 16 mm (range 14 to 19 mm) [2,3,4]. As a result, the ascending aorta (AA) is stretched by 11.6 ± 2.9 mm, while the aortic arch at the level of the brachiocephalic artery and the apex are only displaced by 2.9 ± 0.4 mm (range 0 to 6 mm) [5] and 1.9 ± 0.5 mm (range −0.1 to 5.1 mm), respectively [2,6]. This longitudinal stretching of the AA requires force, which is a direct mechanical load on the LV that may have important implications for the relation between aortic stiffness and LV systolic longitudinal function [7]. Since the progressive deterioration of AA elasticity can hardly be changed, freeing the apex from the pericardial confinement and allowing the apex to move freely towards the base could be an alternative mode of action to aid and restore left ventricular longitudinal shortening. This idea arose from observations during open heart surgery. With a closed pericardium, the base of the heart is drawn towards the cardiac apex. However, once the pericardium is opened at the apical part, the heart’s mode of longitudinal contraction is reversed. Instead of stretching the AA during systolic contraction, the cardiac apex moves towards the heart’s base, drawing air into the pericardial space, but, during diastolic and heart filling, blowing air out of the pericardial space [8]. The present computational analysis was undertaken to mechanistically qualify and quantify the effects of releasing the apex from its pericardial confinement and allowing for inverse longitudinal shortening in a heart with a stiff ascending aorta, to better understand the possible effects on LV mechanics.

## 2. Methods

### Computational Model

We utilized the Living Left Heart Human Model by Dassault Systémes Simulia Corporation (LLHH), which is capable of simulating LV performance, pressure-volume loops, and stress and strain analyses, all of which correlate with clinical observations [9,10]. Our finite element model includes the AA, LV, left atrium, mitral valve, aortic root, and pericardium. The dynamic response is governed by realistic structural and blood flow physics, and the heart contraction is driven by electrical excitation. Blood is represented using a combination of three-dimensional hydrostatic fluid cavities for the heart chambers and system-level chambers to represent arterial and pulmonary compliances. Blood flow occurs inside a closed-loop system between the chambers and the circulatory system through fluid link elements. Details of the model can be found under the “Simulation” and “Virtual Human” sections of Dassault Systémes User Assistance, located at http://help.3ds.com/ (accessed on 11 June 2024).

The passive material response of the cardiac tissue uses an anisotropic hyperelastic formulation proposed by Holzapfel and Ogden, as described in Equation (1) [11].

The passive material parameters were calibrated as follows: the biaxial and triaxial experimental data published by Sommer et al. [12] were used for initial calibration, and diastolic filling tests were used to augment the calibration of the eight material parameters, *a*, *b*, *a_f_*, *b_f_*, *b_s_*, *a_s_*, *b_s_*, *a_n_*, and *b_n_*, which describe the ventricular passive material properties based on the methods described in Klotz et al. [13,14] (Table 1).

(1) $$\Psi_{dev}=\frac{a}{2b}\exp[b(I_{1}-3)]+\sum_{i=f,s}\frac{a_{i}}{{2b}_{i}}\left\{exp\left[b_{i}\left({{(I}_{4i}-1)}^{2}\right)\right]-1\right\}+\frac{a_{fs}}{{2b}_{fs}}[\exp(b_{fs}I_{8fs}^{2}-1)]$$

Equation (1): Passive material response of cardiac tissue. *Ψ_dev_* is the deviatoric strain energy. The parameters *a*, *b*, *a_f_*, *b_f_*, *b_s_*, *a_s_*, *b_s_*, *a_n_*, and *b_n_* describe the ventricular passive material parameters.

The active tissue response contains length-dependent considerations of regional sarcomere lengths, affecting the stress components in the fiber and sheet directions in the constitutive model. The active tissue material model was intended to capture the Frank–Starling effect (i.e., the strength of the heart’s systolic contraction is directly proportional to its diastolic expansion) [15]. The active contraction was simulated by adding stress in the direction of the muscle fiber, defined by a time-varying model of elastance [16] as follows.

Equation (2): T_max_ is the maximum isometric tension achieved at the longest sarcomere length and maximum peak intracellular calcium concentration. T_max_ (N/mm^2^) is a scalar factor representing the maximum active fiber stress or contractility in computational modeling.

(2) $$\sigma_{af}\left(t,E_{ff}\right)=\frac{T_{max}}{2}\frac{{Ca}_{0}^{2}}{{Ca}_{0}^{2}+E{Ca}_{50}^{2}\left(E_{ff}\right)}\left(1-cos\left(\omega \left(t,E_{ff}\right)\right)\right),$$

where

$$E{Ca}_{50}^{2}\left(E_{ff}\right)=\frac{{{Ca}_{0}}_{max}}{\sqrt{e^{B\left(l\left(E_{ff}\right)-l_{0}\right)-1}}}$$

$$\omega \left({t,E}_{ff}\right)=\left\{\begin{array}{c} \pi \frac{t}{t_{0}}\;when\;0\leq t\leq t_{0}\\ \pi \frac{t-t_{0}+t_{r}\left(l\left(E_{ff}\right)\right)}{t_{r}}\;when\;t_{0}\leq t\leq t_{0}+t_{r}\left(l\left(E_{ff}\right)\right)\\ 0\;when\;t\geq t_{0}+t_{r}\left(l\left(E_{ff}\right)\right) \end{array}\right.$$

$$t_{r}\left(l\right)=ml+b$$

$$l\left(E_{ff}\right)=l_{r}\sqrt{2E_{ff}+1}$$

Equation (2): active stress calculation. T_max_ (N/mm^2^) is a scalar factor for myocardial contractility that represents the isometric tension achieved at the longest sarcomere length and maximum peak intracellular calcium concentration. *Ca*_0*max*_ is the peak intercellular calcium concentration. *B* governs the shape of the peak isometric tension and sarcomere length relation. *l*_0_ is the sarcomere length below which no active force develops. *l_r_* is the initial sarcomere length. *t*_0_ is the time to reach the peak tension. *m* and *b* are coefficients that govern the relationship between the linear relaxation duration and sarcomere length relaxation. *E_ff_* is a Lagrangian strain tension component aligned with the local muscle fiber direction [16].

We simulated the mechanical constraints imposed by the pericardium by applying physiological boundary conditions on the ventricular epicardium to achieve the realistic atrioventricular plane motion and radial inward motion of the epicardium, as described in humans [2]. Forty-nine clusters of nodes, evenly distributed on the epicardium surface, were constrained via a spring with higher stiffness when closer to the apex and lower stiffness when closer to the base [17].

The heart was constrained via boundary conditions at the cut planes of the aortic root and pulmonary veins. Each cut plane was constrained relative to a central reference point and the reference point of the pulmonary veins was fixed. The aortic root was constrained from rotation but allowed to stretch. Aortic elasticity was modeled via a spring representing the AA stiffness. The stiffness of the spring was initially set at 0.5 N/mm at baseline to achieve a realistic translation of the proximal aorta of 11.0 mm during systole [18].

The spring stiffness was increased to 10 N/mm to model a stiff AA until a stationary aorta (stationary plane of the sino-tubular junction) was achieved. We performed three simulations under the following conditions: (A) the effect of a mobile AA using the normal AA stiffness as the elastic spring stiffness to constrain the aortic root motion with an amplitude of 11.0 mm; (B) the effect of stiffening the AA by immobilizing the AA at the sino-tubular junction; (C) to model the effect of removing the pericardial confinement at the apex of the heart, the apical boundary conditions of the distal half of the pericardial sack were eliminated in model (B), allowing the free movement of the LV apex. Apex displacement was determined from the coordinates of the epicardial apex and mitral annulus plane center at the maximum length at end-diastole and the minimum length at end-systole and the displacement was computed along this apex–base axis.

Myocardial strain was calculated as the relative length change between the diastolic and the systolic states. The LV strains were measured along the radial, circumferential, and longitudinal directions at 12 locations (three axial and four circumferential locations) at both the epicardium and endocardium. The averages of the tensile strains were reported with positive values. Compressive strains were reported with negative values and depicted as bar graphs. Baseline values were within the reported range of normal human LV strains [19].

The volumetric-averaged myofiber stress was calculated at end-systole in MPa (N/mm^2^) and presented as a contour plot in LV parasternal long-axis cut planes. The left ventricular pressures and volumes were computed and depicted as pressure-volume loops. The area under the pressure-volume loop represents the total effective work (Joule) generated by ventricular contraction, as shown in Equation (3):

(3) SW=SV×MAP

Equation (3): the calculation of the effective stroke work (*SW*) is the area under the pressure-volume loop, *SV* is the stroke volume, and *MAP* is the mean arterial pressure.

## 3. Results

### 3.1. Baseline Simulation

**Figure 1:**
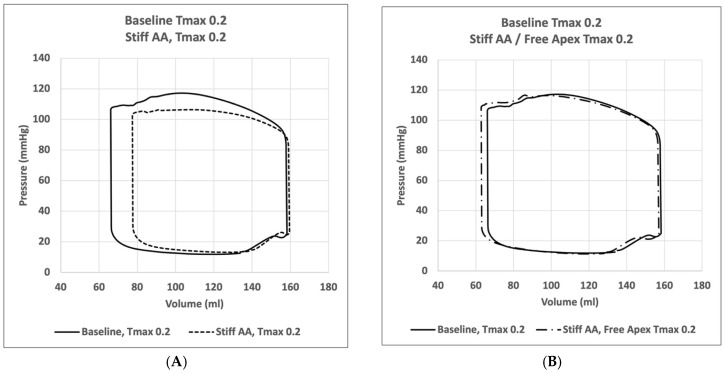
Pressure-volume loops. (**A**) Comparison of pressure-volume loop of left ventricle for simulation with mobile aorta (Baseline) T_max_ 0.2 N/mm^2^ against simulation with stiff AA T_max_ 0.2 N/mm^2^. (**B**) Comparison of pressure-volume loop of left ventricle for simulation with mobile aorta T_max_ 0.2 N/mm^2^ against stiff AA and free apex T_max_ 0.2 N/mm^2^.

**Figure 2:**
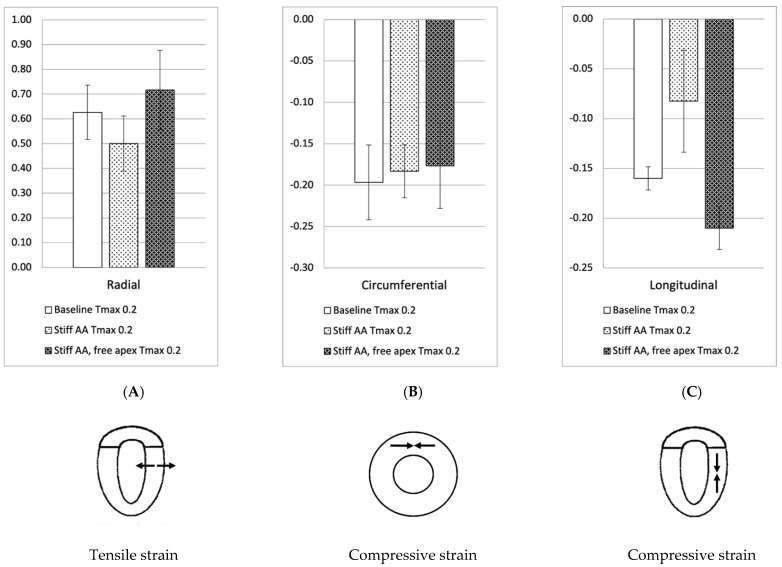
Left ventricular strains. Left ventricular strain for baseline simulation T_max_ 0.2 N/mm^2^, simulation with stiff AA T_max_ 0.2 N/mm^2^, and simulation with stiff AA and free apex T_max_ 0.2 N/mm^2^. (**A**) Radial strain (three radial locations) is depicted as positive with wall thickening from diastole to systole. (**B**) Circumferential strain (three circumferential locations) is depicted as negative when circumference is reduced from diastole to systole. (**C**) Longitudinal strain (four longitudinal locations) is depicted as negative when apex base length is reduced from diastole to systole.

**Figure 3:**
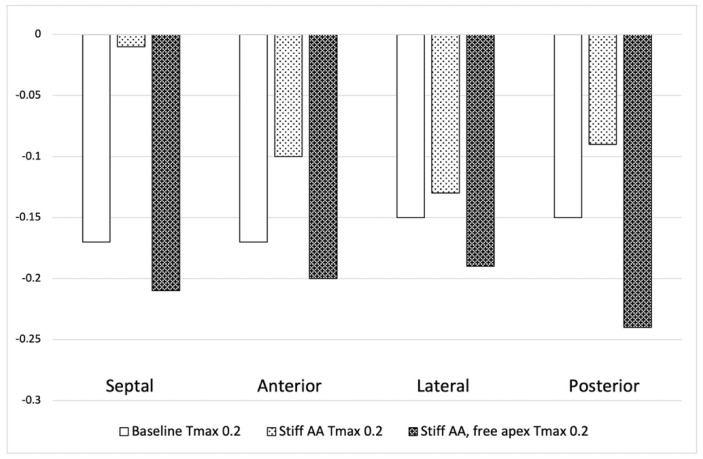
Left ventricular longitudinal strain in four longitudinal regions (septal, anterior, lateral, and posterior strain) at baseline T_max_ 0.2 N/mm^2^, stiff AA T_max_ 0.2 N/mm^2^, and stiff AA with free apex T_max_ 0.2 N/mm^2^.

In the initial simulation (model A), with contractility T_max_ of 0.2 N/mm^2^, the aortic root underwent 11.0 mm displacement towards the apex during systole, whereas the apex moved 1.9 mm in the opposite direction [2]. The stroke volume and stroke work calculated in this baseline simulation were 92.2 mL and 8747 Joules, respectively (Table 2). A pressure-volume loop was generated, demonstrating the expected pattern (Figure 1A). The LV strain profiles at the end-systole are depicted in Figure 2 and Figure 3 and Table 3. Throughout systolic contraction, the wall exhibited thickening with a radial strain of 0.63 ± 0.11, while the circumference displayed a reduction with a circumferential strain of −0.20 ± 0.05 (Table 3, Figure 2). The average apex base length shortened with an average longitudinal strain of −0.16 ± 0.01 (Table 3 and Table 4, Figure 3). The calculated average myofiber stress was 0.056 ± 0.036 MPa (Table 5).

A contour plot depicting the systolic regional stress distribution at end-systole is shown inFigure 4A. Notably, the areas with the highest myofiber stress appeared at the mitral annulus, the fibrous trigones, the aorto-mitral junction, and the papillary muscle tip.

Computational simulation of LV pressure and volume, stroke volume, and effective stroke work at baseline T_max_ 0.2 N/mm^2^, stiff AA T_max_ 0.2 N/mm^2^, and stiff AA with free apex T_max_ 0.2 N/mm^2^. (EDP: end-diastolic pressure; EDV: end-diastolic volume; ESP: end-systolic pressure; ESV: end-systolic volume; SVed-es: stroke volume; SW: stroke work).

### 3.2. Effect of Stiff Ascending Aorta

**Figure 4:**
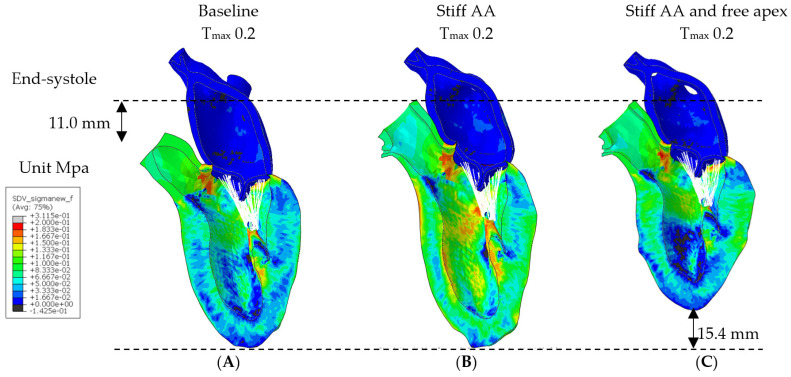
Myofiber stress. Long-axis profile of LV at end of systole showing contours of myofiber stress at end-systole. (**A**) Baseline T_max_ 0.2 N/mm^2^, (**B**) stiff AA T_max_ 0.2 N/mm^2^, and (**C**) stiff AA and free apex T_max_ 0.2 N/mm^2^. Dotted line indicates baseline level of ascending aorta at end-diastole and level of apex.

**Figure 5:**
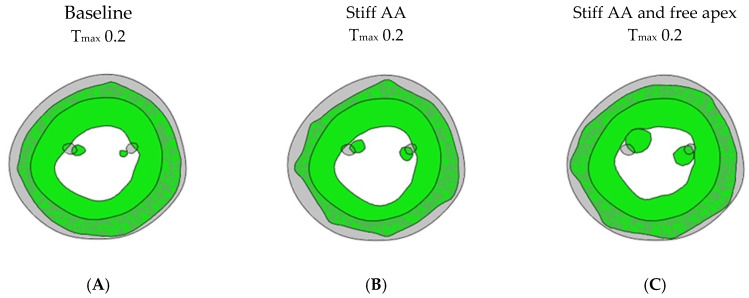
Cross-sectional LV profile. Cross-sectional profile of LV at center of longitudinal LV axis and base of papillary muscles. Grey color showing end-diastolic shape at baseline; green color showing end-systolic shape. (**A**) Baseline T_max_ 0.2 N/mm^2^, diameter 59 mm; (**B**) stiff AA T_max_ 0.2 N/mm^2^, diameter 57 mm; and (**C**) stiff AA and free apex T_max_ 0.2 N/mm^2^, diameter 63 mm.

In model (B) with baseline contractility (T_max_ 0.2 N/mm^2^) and with a stiff ascending aorta, the sino-tubular junction as well as the LV apex were stationary (Figure 4B). At the level of the papillary muscle tip along the longitudinal axis of the LV, the transverse end-systolic diameter decreased from 59 mm to 57 mm, representing a reduction of 3.4% from the baseline value (Figure 5). The cross-sectional profile of the LV shape demonstrated that the LV tended to be more ovalized at end-systole.

Compared to the baseline measurements, the analysis of the pressure-volume loop (Figure 1A) demonstrated that the end-diastolic pressure increased by 8.5% and the end-systolic pressure showed a reduction of 9.1%. While the end-diastolic volume remained nearly unchanged (1.3%), the end-systolic volume increased by 17.1%; consequently, the stroke volume was decreased by 10.9% and the effective stroke work was reduced by 19.0% (Table 2). The analysis of the pressure-volume loop showed a corresponding decrease in the end-systolic LV pressure and an increase in the end-diastolic volume (Figure 1A).

The LV strain profiles at end-systole, along with their respective values (Table 3), are depicted inFigure 2 and Figure 3. The average radial strain, the circumferential strain, and the longitudinal stain displayed a reduction of 20.2 ± 2.4%, 6.8 ± 10.9%, and 48.4 ± 36.9%, respectively (Table 3, Figure 2). While the septal longitudinal strain was reduced the most, by 94.1%, the anterior, lateral, and posterior strain measures were reduced by 41.2%, 13.3%, and 40.0%, respectively, indicating that AA stiffening exerts the greatest effect upon the septal longitudinal stain (Table 4, Figure 3). The average myofiber stress increased by 36.98 ± 42.91% in comparison to the baseline from 0.056 ± 0.036 to 0.076 ± 0.042 MPa (Table 5). The systolic regional stress distribution at end-systole is presented as a contour plot in Figure 4B. Stress increased overall in the LV, with very high-stress areas noted at the septum, the papillary muscles, the mitral annulus, the fibrous trigones, and the aorto-mitral junction.

### 3.3. Effect of Removing Pericardial Boundary Conditions at Distal Half of Pericardial Sack with Stiff Ascending Aorta

In model C, with a stiff AA, the pericardial boundary conditions were eliminated in the distal half of the pericardial sac, allowing for the unrestricted movement of the LV apex. Systolic contraction with the same LV passive and active tissue properties and with the same contractility T_max_ of 0.2 N/mm^2^ as in the previous models (A) and (B) caused the LV apex to move during systole towards the base of the heart by 15.4 mm (Figure 4C).

Compared to the baseline measurements, the analysis of the pressure-volume loop (Figure 1B) demonstrated that the end-diastolic pressure, which was increased with the stiffening of the AA by 8.5%, dropped 5.1% below the baseline value. The end-systolic pressure, which was reduced by 9.1% with the stiffening of the AA, returned to 0.43% below the baseline values. After the stiffening of the AA, the stroke volume and effective stroke work decreased by 10.9% and 19.0%, respectively. However, upon allowing for the unrestricted movement of the LV apex, these values returned to 2.1% and 2.0% above their respective baseline values (Table 2), and the overall visual aspect of the pressure-volume loop returned to the baseline shape (Figure 1B). The transverse end-systolic diameter at the center of the longitudinal LV axis at the tip of the papillary muscles increased to 63 mm, or 6.8% over the baseline values (Figure 5C). The cross-sectional profile of the LV demonstrated the increasing globalization of the LV shape. The average radial and longitudinal strains increased by 14.4 ± 9.7% and 31.3 ± 16.9%, respectively, while the circumferential strain remained reduced by 10.2 ± 18.3% below the baseline values (Table 3, Figure 2). All reginal longitudinal strains recovered, with the septal, anterior, lateral, and posterior strains being 23.5%, 17.7%, 26.7%, and 60.0% over the baseline values (Table 4, Figure 3).

The calculated average myofiber stress, which was increased with the stiffening of the AA to 0.076 ± 0.042 MPa, or 36.98 ± 42.91% over the baseline values, deceased by 67.5% to 0.062 ± 0.038 MPa or 12.0 ± 42.2% over the baseline values (Table 5). The systolic stress decreased overall in the LV, with remarkably reduced stress areas along the septum and the LV lateral wall, the papillary muscles, and the apex (Figure 4C).

## 4. Discussion

Throughout the cardiac cycle, the heart’s epicardial apex remains stationary within the fluid-tight pericardial sac [1], which is anchored to the diaphragm. The pericardial sac’s apex is also connected to the caudal sternum by the sterno-pericardial ligament, effectively linking the caudal sternum to the LV apex. This creates a relatively straight line of force that runs from the stationary LV apex at the caudal end to the stationary aortic arch [5] at the cranial end, with the elastic AA situated in between. During the cardiac cycle, as the LV contracts longitudinally and the AA is stretched, the aortic root moves up and down along this line of force. It is well recognized that AA stiffening increases with age [7,20,21,22]. Numerous mechanisms have been suggested to account for this phenomenon, such as alterations in endothelial function, modifications in the compositions of structural proteins, collagen crosslinking, alterations in vascular geometry, and neurohumoral signaling [20,23,24]. Aortic stiffening is a well-known cause of reduced arterial compliance (i.e., elasticity) and impaired aorto-ventricular interaction. This can lead to a significant reduction in longitudinal left ventricular function with the reduced descent of the atrio-ventricular plane during systole and a decreased average long-axis strain, the impaired early diastolic filling of the ventricle, a higher left ventricular afterload, and a higher end-diastolic LV pressure [7,25,26,27]. With the stiffening and reduced longitudinal elasticity of the aortic root, a higher load on the oblong-oriented myocardial fibers is expected and a stiff AA would be stretched and displaced less than a compliant aorta. The heart would have to contract with a greater long-axis force to produce the same amount of aortic displacement and, consequently, the same stroke volume [6]. Studies in animals have demonstrated that a stiff aorta can cause a significant 30% increase in myocardial oxygen consumption and a 20–40% increase in the energy required by the heart to deliver a given stroke volume [28,29]. Similarly, clinical studies in humans have shown that arterial stiffening raises myocardial oxygen consumption by over 50% for a given stroke volume [29]. The authors have concluded that although aortic stiffening may not have an impact on heart function at rest, it can limit the reserve capacity under conditions of increased demand [28]. It was demonstrated in humans that increased aortic stiffness is associated with a reduction in global longitudinal strain, which supports the hypothesis that aortic stiffening imposes a direct mechanical load on long-axis LV function [7,20,30,31,32]. With aortic stiffening, the force required to stretch the ascending aorta increases, resulting in an increased load on the long axis of the LV and eventually leading to a decrease in LV long-axis shortening [6].

In the present computational study, the stiffening and reduced longitudinal elasticity of the aortic root caused a greater load on the longitudinal myocardial fibers, with increased myocardial stress (+37.0%), which led to reduced average LV longitudinal (−20.2%) and especially septal longitudinal strain (−94.1%) and the subsequent deterioration of longitudinal LV function with decreased effective stroke work (−19.0%) and increased end-diastolic pressure (+8.5) compared to baseline values. The reduced longitudinal elasticity of the AA affects longitudinal myocardial shortening, thus posing an additional load and stress upon the myofibers. This further adversely affects LV function, predisposing patients towards HFpEF syndrome [29,30,31,32,33]. HFpEF is associated with significantly impaired LV global longitudinal strain [25,34,35] and longitudinal LV systolic function [29,33,36,37,38]. At the same time, 65% of HFpEF patients harbor AA stiffening (beyond age-associated values) [35]. The fact that HFpEF symptoms strongly correlate with increased arterial stiffness [7,20,30,31,32] suggests a possible pathophysiologic link between aortic stiffness, reduced AA stretching, decreased atrioventricular plane displacement, and alterations in LV systolic longitudinal function, contributing to the pathophysiology of HFpEF syndrome [7,30,35]. Consequently, arterial stiffness has been proposed as a potential causative factor leading to HFpEF [7,30] and might be the pathologic mechanism that drives the progression from diastolic dysfunction to HFpEF [33].

Healthy women have a shorter AA at 79 mm vs. 86 mm and significantly greater AA longitudinal strain than men (8.5% vs. 6.7%) [6]. Postmenopausal women tend to have higher arterial stiffness as compared to men [39]. Within one year of the final menstrual period, women experience a rapid and significant increase in aortic stiffening independent of age [39], where black women have a greater increase in arterial stiffness than white women [40]. While the development of diastolic dysfunction of the left ventricle is equally common in women and men, women outnumber men with HFpEF by a 2:1 ratio [41,42], and women with HFpEF tend to show a poorer prognosis, including lower quality of life as compared to men [42]. The shorter AA with greater AA longitudinal strain and the increased tendency for AA stiffening in women may create earlier and larger myocardial stress as compared to men and explain in part why women may be more predisposed to HFpEF [41].

The prevailing dogma that heart failure is an irreversible disease has been challenged by observations in some heart failure patients with myocardial recovery post-LVAD, offering support to the hypothesis of a mechanically exhausted myocardium that has the potential to recover [43,44,45]. This phenomenon has also been observed in reversible exercise-induced cardiac fatigue [46,47,48]. Novel mechano-energetic concepts propose myocardial fatigue with impaired contractility and relaxation in the face of adverse loads, particularly caused by a stiffened arterial system [43,44,49,50]. Like the skeletal muscles, a fatigued myocardium, as proposed in HFpEF, is largely structurally normal and should have the potential to recover, as long as its myocytes can be mechanically unloaded (e.g., with arterial vasodilators or left ventricular assistance devices in selected cases). In our in silico study, we mechanically unloaded the LV by removing the boundary condition of the pericardium at the apical part (Figure 4C), allowing the apex to become mobile. The apex moved to the heart’s base by 15.4 mm, instead of stretching the AA (11.0 mm at baseline), achieving inverse longitudinal shortening. Beforehand, the reduced radial and longitudinal strain recovered beyond the baseline, with overall reduced myocardial stress (−18.4%), increased effective stroke work (+26.0%) and stroke volume (+12.7%), and reduced end-diastolic pressure (−12.5%) compared to model B with the stiff aorta. Freeing the apex from the pericardial confinement and allowing the apex to move freely made it easier for the simulated heart to shorten. With this recovered LV function, an increased stroke volume and reduced end-diastolic pressure were achieved.

**Figure 6:**
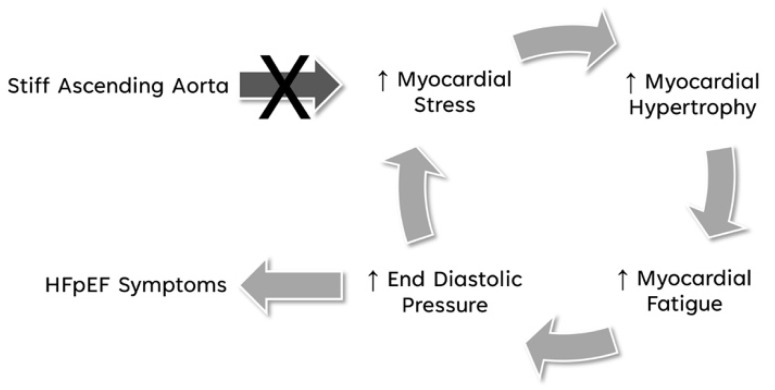
Breaking the vicious cycle of stiffened AA to HFpEF symptoms. Eliminating the high myocardial load created by a stiff AA by freeing the apex from its pericardial constraint has the potential to break the vicious cycle of increased myocardial fiber stress, reactive myocardial hypertrophy, subsequent myocardial fatigue, and rising ventricular end-diastolic/left atrial pressure, leading to HFpEF symptoms and further increasing the myocardial fiber stress.

We hypothesize that eliminating the high myocardial load created by a stiff AA by freeing the apex from its pericardial constraint has the potential to break the vicious cycle of increased myocardial fiber stress, reactive myocardial hypertrophy, subsequent myocardial fatigue, and rising ventricular end-diastolic/left atrial pressure, further increasing the myocardial fiber stress (Figure 6), and will prevent the transition to irreversible myocardial damage, where prolonged fatigue and ongoing inflammation may lead to myocardial fibrosis [35,43,44,49,51].

We propose a novel mode of action for the treatment of HFpEF syndrome associated with a stiff AA. Freeing the apex from the pericardial confinement and allowing the apex to move freely towards the base, could be an alternative approach to reduce the heart’s mechanical load, making it easier for the heart to shorten, to restore LV function, and to recover from myocardial fatigue.

The effect of opening the pericardium and freeing the apex was studied in HFpEF patients who underwent open heart surgery. The opening of the pericardium attenuated the increase in LV filling pressures that develops during volume loading in humans with HFpEF, demonstrating a potential therapeutic opportunity in HFpEF patients [52]. Freeing the apex by pericardiotomy alone has an immediate effect but will also create adhesions, which, within a short period, again restrict the movement of the cardiac apex [53,54,55]. The illicit use of pericardiotomy to improve racing results in greyhound dogs has been described, but the effect vanished after the occurrence of adhesions [53,56]. We, therefore, propose the implantation of a passive pressure decompensation chamber that provides a fluid volume in the pericardial space in systole, allowing the apex to move towards the heart’s base, and removes such volume from the pericardial space in diastole, allowing the apex to move away from the heart’s base for cardiac filling.

## 5. Limitation of Study

Although the Living Left Heart Human (LLHH) Model has seen considerable use in cardiac modeling, several limitations apply [9,15]. The LLHH model includes the aortic arch, left ventricle, left atrium, mitral valve, aortic root, and pericardium, while the right heart is not captured in the model. As a result, potential effects or influences of these structures on the left heart are not accounted for. Furthermore, the material properties of the ascending aorta remain unaltered, and the stiff aorta is simulated by immobilizing the arch at the level of the sino-tubular junction, which may not fully simulate the mechanics of a stiffened aortic wall. In the case of a stiff aorta, the sympathetic nerves and humoral regulation are expected to increase the myocardial contractility to restore a normal cardiac output. However, hemodynamic feedback control is not modeled to automatically regulate myocardial contractility to maintain the cardiac output. Instead, the contractility is uniformly increased in all myocytes, neglecting the possible anisotropy and remodeling of the left ventricle. The noticeable surface irregularities (Figure 3 and Figure 4) are a result of the simplified representation of the pericardium, achieved through springs connected to forty-nine clusters of nodes evenly distributed on the epicardium surface. Employing more nodes with spring stiffness inversely proportional to the displacement could potentially lead to a smoother surface but would not affect the overall results [17]. In summary, the LLHH model is a valuable tool for an understanding of the impact of the stiffening of the ascending aorta on left ventricular function. However, it is essential to acknowledge its limitations, including the omission of certain heart structures, assumptions about material properties, and the absence of hemodynamic feedback control and a simplified pericardium when interpreting the simulation results.

## 6. Conclusions

The findings of this study conducted in silico highlight the significant pathophysiological relationship between a stiff AA and reduced longitudinal strain, contributing to the deterioration of longitudinal LV systolic function, often observed in patients suffering from heart failure with preserved ejection fraction (HFpEF). The simulations reveal that the stiffening of the AA causes an increase in the end-diastolic filling pressure and a decrease in the end-systolic pressure, along with reductions in the stroke volume and effective stroke work. In addition, the average radial, circumferential, and longitudinal strains showed marked reductions, while the average myofiber stress increased, indicating potential deleterious effects on LV function, potentially leading to hypothesized myocardial fatigue. This in silico study introduces a novel, theoretical therapeutic approach by suggesting that releasing the LV apex to enable inverse LV longitudinal shortening could mitigate the adverse mechanical effects induced by a stiff AA. The simulation of this condition showed promising results, with improvements in the end-diastolic and end-systolic pressures, stroke volume, and effective stroke work, compared to the stiff AA scenario. Additionally, the average radial and longitudinal strains increased. Most notably, the calculated average myofiber stress was significantly reduced, potentially allowing for recovery from myocardial fatigue.

The stiffness of the AA and impairment of LV longitudinal strain are common in patients with HFpEF. Therefore, the promising results of this hypothesis-generating study provide a new direction for future experimental and clinical research aimed at new treatment options for this patient group. Pre-clinical and clinical studies are required to validate the proposed approach.

## Figures and Tables

**Table 1:** Constructive parameters for the passive and active material response.

Passive Parameters										
	*a* (MPa)	*b*	*a_f_* (MPa)	*b_f_*	*a_s_* (MPa)	*b_s_*	*a_fs_* (MPa)	*b_fs_*		Calibration Data
Atrium	1.0 × 10^−3^	3.1	4.7 × 10^−3^	1.2 × 10^+1^	2.7 × 10^−3^	9.1	9.0 × 10^−7^	6.7 × 10^−4^		Sommer [12], Klotz [13]
Ventricle	3.9 × 10^−4^	3.7	1.9 × 10^−3^	1.4 × 10^+1^	1.1 × 10^−3^	1.1 × 10^+1^	3.6 × 10^−7^	7 × 10^−4^		Sommer [12], Klotz [13]
**Active Parameters**										
	***t_0_*** **(s)**	***m*** **(s/mm)**	**b** **(s)**	**l_0_** **(mm)**	***B*** **(1/mm)**	***Ca_0max_*** **(mM)**	***Ca_0_*** **(mM)**	***T_max_*** **(Mpa)**	***L_r_*** **(mm)**	**Reference**
Atrium	0.05	1048.9	−1.5	0.00158	4750	4.35	4.35	0.1	0.00185	Sack [15], Guccione [14]
Ventricle	0.35	950	−1.5	0.00158	4750	4.35	4.35	0.2	0.00185	Sack [15], Guccione [14]

**Table 2:** LV pressure and volume.

	EDP	EDV	ESP	ESV	SVed-es	SW
	(mmHg)	(mL)	(mmHg)	(mL)	(mL)	(Joule)
Baseline T_max_ 0.2	11.85	158.30	117.10	66.10	92.20	8747.50
Stiff AA T_max_ 0.2	12.86	159.60	106.40	77.40	82.20	7084.50
Stiff AA with free apex T_max_ 0.2	11.25	157.00	116.60	62.86	94.14	8923.00
Baseline vs. stiff AA T_max_ 0.2 vs. T_max_ 0.2	1.01	1.30	−10.70	11.30	−10.00	−1663.00
Baseline vs. stiff AA T_max_ 0.2 vs. T_max_ 0.2 (%)	8.52%	0.82%	−9.14%	17.10%	−10.85%	−19.01%
Baseline vs. stiff AA with free apex T_max_ 0.2 vs. T_max_ 0.2	−0.60	−11.3	−0.50	−3.24	1.94	175.50
Baseline vs. stiff AA with free apex T_max_ 0.2 vs. T_max_ 0.2 (%)	−5.06%	−0.82%	0.43%	−4.90%	2.10%	2.01%

**Table 3:** Average strain.

Average Strain	Radial	Circumferential	Longitudinal
Baseline T_max_ 0.2	0.63 ± 0.11	−0.20 ± 0.05	−0.16 ± 0.01
Stiff AA T_max_ 0.2	0.50 ± 0.11	−0.18 ± 0.03	−0.08 ± 0.05
Stiff AA and free apex T_max_ 0.2	0.72 ± 0.16	−0.18 ± 0.05	−0.21 ± 0.02
Baseline T_max_ 0.2 vs. stiff AA T_max_ 0.2	−0.13 ± 0.02	0.01 ± 0.02	0.08 ± 0.06
Baseline T_max_ 0.2 vs. stiff AA T_max_ 0.2 (%)	−20.21 ± 2.39%	−6.78 ± 10.86%	−48.44 ± 36.88%
Baseline T_max_ 0.2 vs. stiff AA and free apex T_max_ 0.2	0.09 ± 0.06	0.02 ± 0.04	−0.05 ± 0.03
Baseline T_max_ 0.2 vs. stiff AA and free apex T_max_ 0.2 (%)	14.36 ± 9.73%	−10.17 ± 18.31%	31.25 ± 16.88%

Average radial, circumferential, and longitudinal strain at baseline T_max_ 0.2 N/mm^2^, stiff AA T_max_ 0.2 N/mm^2^, and stiff AA with free apex T_max_ 0.2 N/mm^2^.

**Table 4:** Longitudinal strain.

Longitudinal Strain	Septal	Anterior	Lateral	Posterior
Baseline T_max_ 0.2	−0.17	−0.17	−0.15	−0.15
Stiff AA T_max_ 0.2	−0.01	−0.10	−0.13	−0.09
Stiff AA and free apex T_max_ 0.2	−0.21	−0.2	−0.19	−0.24
Baseline T_max_ 0.2 vs. stiff AA T_max_ 0.2	0.16	0.07	0.02	0.06
Baseline T_max_ 0.2 vs. stiff AA T_max_ 0.2 (%)	−94.12%	−41.18%	−13.33%	−40.00%
Baseline T_max_ 0.2 vs. stiff AA and free apex T_max_ 0.2	−0.04	−0.03	−0.04	−0.09
Baseline T_max_ 0.2 vs. stiff AA and free apex T_max_ 0.2 (%)	23.53%	17.65%	26.67%	60.00%

Longitudinal strain in four regions, septal, anterior, lateral, and posterior, at baseline T_max_ 0.2 N/mm^2^, for stiff AA T_max_ 0.2 N/mm^2^, and for stiff AA with free apex and T_max_ 0.2 N/mm^2^.

**Table 5:** Myofiber stress.

Stress	Baseline	Stiff AA	Stiff AA and Free Apex
	T_max_ 0.2	T_max_ 0.2	T_max_ 0.2
(MPa)	0.056 ± 0.036	0.076 ± 0.042	0.062 ± 0.038
vs. Baseline		36.98 ± 42.91%	12.03 ± 42.19%

Average myofiber stress at baseline T_max_ 0.2 N/mm^2^, stiff AA T_max_ 0.2 N/mm^2^, and stiff AA with free apex T_max_ 0.2 N/mm^2^. Comparison with baseline.

## Data Availability

Data are available from the corresponding author upon reasonable request. Restrictions apply to the availability of some data, which were used under license for this study.
